# Significance of *Streptococcus gallolyticus* subsp. *gallolyticus* Association With Colorectal Cancer

**DOI:** 10.3389/fmicb.2018.00614

**Published:** 2018-04-03

**Authors:** Ewa Pasquereau-Kotula, Mariana Martins, Laetitia Aymeric, Shaynoor Dramsi

**Affiliations:** ^1^Unité de Biologie des Bactéries Pathogènes à Gram-Positif, Institut Pasteur, Paris, France; ^2^Unité de Pathogénie Microbienne Moléculaire, Institut Pasteur, Paris, France

**Keywords:** *S. gallolyticus*, colorectal cancer, infective endocarditis, gut colonization, pili

## Abstract

*Streptococcus gallolyticus* subsp. *gallolyticus Sgg* (formerly known as *S. bovis* type I) is the main causative agent of septicemia and infective endocarditis (IE) in elderly and immunocompromised persons. It belongs to the few opportunistic bacteria, which have been strongly associated to colorectal cancer (CRC). A literature survey covering a period of 40 years (1970–2010) revealed that 65% of patients diagnosed with an invasive *Sgg* infection had a concomitant colorectal neoplasia. *Sgg* is associated mainly with early adenomas and may thus constitute an early marker for CRC screening. *Sgg* has been described as a normal inhabitant of the rumen of herbivores and in the digestive tract of birds. It is more rarely detected in human intestinal tract (2.5–15%). Recent molecular analyses indicate possible zoonotic transmission of *Sgg*. Thanks to the development of a genetic toolbox and to comparative genomics, a number of factors that are important for *Sgg* pathogenicity have been identified. This review will highlight the role of *Sgg* pili in host colonization and how their phase-variable expression contributes to mitigate the host immune responses and finally their use as serological diagnostic tool. We will then present experimental data addressing the core question whether *Sgg* is a cause or consequence of CRC. We will discuss a few recent studies examining the etiological versus non-etiological participation of *Sgg* in colorectal cancer with the underlying mechanisms.

## Introduction

*Streptococcus gallolyticus* belongs to the Group D streptococci, a large group of phenotypically diverse bacteria known as the *S. bovis*/*S. equinus* complex (SBSEC), which consist of safe-graded bacteria used in food-fermentation, commensal bacteria of the gut and opportunistic pathogens in both humans and animals. About 15 years ago, a revised classification of this streptococcal group has been proposed (Poyart et al., 2002; Schlegel et al., 2003), but the new taxonomy is still not completely adopted by the scientific community, especially the clinicians, resulting in confusing names in the literature. The currently admitted classification based on multilocus sequence typing (MLST) data defines seven subspecies: *Streptococcus gallolyticus* subsp. *gallolyticus* (*Sgg*), *S. gallolyticus* subsp. *macedonicus* (*Sgm*), *S. gallolyticus* subsp. *pasteurianus* (*Sgp*), *Streptococcus infantarius* subsp. *infantarius* (*Sii*), *Streptococcus lutetiensis, Streptococcus alactolyticus* and *Streptococcus equinus* (Jans et al., 2015). *Sgg* is an opportunistic pathogen causing septicemia and endocarditis in elderly persons. Intriguingly, several clinical studies have demonstrated a strong association between invasive infections with *Sgg* and colon neoplasia in humans (Boleij et al., 2011). Colorectal cancer (CRC) is firstly a genetic disease that develop over several years involving a series of genetic changes (i.e., somatic mutations and epigenetic modifications) known as the adenoma-carcinoma sequence. Emerging studies have closely linked CRC development with gut microbiota changes (Braten et al., 2017; Flemer et al., 2017; Gao et al., 2017; Lucas et al., 2017). Earlier studies indicated a strong correlation between CRC and intestinal colonization by single bacterial species, such as colibactin-producing *Escherichia coli*, toxin-producing *Bacteroides fragilis, Fusobacterium* spp. and *Streptococcus gallolyticus* subspecies *gallolyticus* (*Sgg*). *Sgg* is commonly found in the flora of herbivores’ rumen, and therefore transmission from animal to human is highly suspected. In this review, we will discuss the specific traits that make *Sgg* a successful opportunistic pathogen in humans. We will then ask the million-dollar question whether *Sgg* is a cause or consequence of CRC. Recent evidence supporting the oncogenic role of *Sgg* but also evidences supporting the beneficial role of tumor microenvironment for *Sgg* outgrowth will be discussed.

### *S. gallolyticus* subsp. *gallolyticus* (*Sgg*) Is an Opportunistic Pathogen

*Sgg* is a normal inhabitant of the gastrointestinal tract of different mammalian herbivores and birds. This bacterium was first isolated from Koala feces most probably because *Sgg* is able to degrade tannins, which are highly polar polyphenolic molecules, present in high quantity in eucalyptus leaves (Osawa, 1990). *S. gallolyticus* owes its name to its capacity to decarboxylate gallate, an organic acid derived from tannins hydrolysis (Osawa and Sasaki, 2004). *S. gallolyticus* can also be found outside the animal host as a saprophyte (Chamkha et al., 2002; Braten et al., 2017; Flemer et al., 2017; Gao et al., 2017; Lucas et al., 2017). *Sgg* was also detected in the human intestinal tract, but remains a low-abundance species (2.5–15%). However, a recent study conducted in Germany using sensitive PCR technique to detect *Sgg* indicated a higher carriage rate estimated at 62.5% in the stools of 99 healthy volunteers (Dumke et al., 2017). Rural residency and animal contact were shown to increase the detection rate of *Sgg* in humans further supporting the zoonotic potential of this bacterium. Indeed, the first complete genome of *Sgg* has provided several insights into the adaptation of *Sgg* to the rumen of herbivores and its capacity to cause endocarditis (Rusniok et al., 2010). In particular, it revealed the existence of many genes involved in plant carbohydrates degradation, two genes encoding tannases and a gene encoding a bile salt hydrolase conferring the bacterium the ability to survive in the gut. Metabolic pathways analysis indicates that *Sgg* should be able to synthesize all 20 amino-acids and most vitamins, thus displaying few nutritional requirements (Rusniok et al., 2010). Similar to other pathogenic streptococci, *Sgg* encodes an extracellular capsule exhibiting a high degree of similarity to *S. pneumoniae* serotype 23F. It was proposed that the polysaccharide capsule protects Sgg from the host innate immune responses, blocking for example pro-inflammatory IL-8 response in epithelial cells (Boleij et al., 2011). Surface proteins of pathogenic bacteria are often involved in the colonization of host tissues. Three pilus loci were revealed and further studied. To analyze the role of these pili, a genetic toolbox was developed enabling inactivation or overexpression of specific genes in *Sgg* UCN34 (Danne et al., 2013).

### Colonization of Host Tissues by *S. gallolyticus* subsp. *gallolyticus*

Like their counterpart in Gram-negative bacteria, Gram-positive pili have often been associated with bacterial attachment and colonization of the host tissues (Danne and Dramsi, 2012). In Sgg, pili were first revealed by ultrastructural studies from pigeons virulent strains (Vanrobaeys et al., 1999). Molecular evidence was provided a decade later in strain TX20005 when a genome-wide analysis revealed the presence of three putative pilus operons (Sillanpaa et al., 2009). The first complete genome of another *Sgg* clinical isolate UCN34 confirmed the presence of three pilus operons, namely *pil1, pil2* and *pil3* (Rusniok et al., 2010). Both *pil1* and *pil3* are highly conserved loci among sequenced *Sgg* strains, whereas *pil2* appears more variable (Sillanpaa et al., 2009; Rusniok et al., 2010). The first virulence factor characterized in *Sgg* was the Pil1 pilus. This pilus was shown to mediate *Sgg* binding to collagen types I and IV and in the bacterial attachment to the heart valves, thereby initiating endocarditis development (Danne et al., 2011). The Pil1-associated adhesin was shown to bind to various types of collagen with different affinities (Sillanpaa et al., 2009). More recently, the Pil1 adhesin was shown to bind to blood factor XII with a very high affinity, leading to activation of human contact system, which in turn results in prolongation of the coagulation time (Isenring et al., 2017). Manipulation of the host coagulation system by *Sgg* is proposed to contribute to virulence. Interestingly, Sgg isolates causing septicemia in pigeons are not able to bind to collagen type I (Vanrobaeys et al., 2000). While collagen type I is the major structural component of human heart, collagen type IV is found in the basal lamina layer underneath epithelial tissue. It is worth pointing out that colonic tumors display higher levels of collagen IV compared to normal tissues (Skovbjerg et al., 2009), which may explain a higher colonization of tumor sites by *Sgg*.

Next, it was shown that the Pil3 pilus was involved in *Sgg* binding to colonic mucus and thus promotes colonization of murine distal colon (Skovbjerg et al., 2009; Martins et al., 2015, 2016). By immunofluorescence on intestinal tissues following mice infection, *Sgg* was mainly found entrapped in the mucus layer (Skovbjerg et al., 2009; Martins et al., 2015, 2016). The Pil3A adhesin was shown to bind to MUC2 and MUC5AC mucins (Skovbjerg et al., 2009; Martins et al., 2015, 2016). MUC2 mucin is a major constituent of the adhesin mucus, whereas MUC5AC is not detected under normal circumstances. Importantly, aberrant and mislocalized expression of MUC5AC mucin in adenomas and carcinomas has been reported (Bartman et al., 1999; Sylvester et al., 2001; Bryant and Stow, 2004), as well as modification of mucins glycosylation patterns during colonic carcinogenesis (Devine and McKenzie, 1992; Itzkowitz et al., 1992; Mann et al., 1997; Jenab et al., 2001; Mesquita et al., 2003).

Pil1 and Pil3 pilus are expressed heterogeneously in *Sgg* UCN34 population (Danne et al., 2014). In UCN34, two distinct sub-populations were found: two-third of low-piliated bacteria (Pil^Low^), and one third of high-piliated bacteria (Pil^high^). The molecular mechanism involved in this regulation has been identified as a combination of phase variation and transcriptional attenuation (Danne et al., 2014). Genetic evidence demonstrated that this heterogeneous expression is dependent on changes in the *pil1* promoter region which includes a leader peptide composed of a variable number of GCAGA repeats followed by a transcription terminator. Addition or deletion of a single repeat by slip-strand mispairing during replication modifies the length of the regulatory leader peptide to be translated. The synthesis of a longer leader peptide controls the switch of pilus transcription through a destabilization of a stem-loop transcription terminator upstream of *pil1* genes (Danne et al., 2014). Hyper-piliated bacteria were found more prone to phagocytosis by human macrophages and had a lower rate of survival in human blood compared to bacteria expressing low levels of pili (Danne et al., 2014). Thus, it was proposed that stochastic expression of the pilus in *Sgg* is an advantageous bacterial feature insuring an optimal tissue colonization and dissemination while evading the host immune responses.

Pilus components of pathogenic streptococci were shown to be promising vaccine candidates because they can induce protective immunity in mouse models (Margarit et al., 2009; Soriani and Telford, 2010). Since pili are often highly immunogenic surface appendages, detection of specific anti-pilins IgG may constitute an ideal serological diagnostic tool that could help in discriminating patients with early adenomas from healthy people. A small proof of concept study combining four pilus proteins demonstrated some potential for this approach (Boleij et al., 2012b). Using a larger cohort (576 CRC cases and 576 controls matched by sex, age and providence), it was shown that only 14% of CRC patients displayed *Sgg-*specific IgG antibodies (Butt et al., 2016). Detection of *Sgg* presence by measuring mucosal IgA antibodies may increase the sensitivity of this test.

### Colorectal Cancer and Microbiota

Colorectal cancer (CRC) is one of the most commonly diagnosed tumors with a high mortality rate (Ferlay et al., 2015). The global burden of CRC is expected to increase by 60% to more than 2.2 million new cases and 1.1 million deaths by 2030 (Arnold et al., 2017). The majority of CRC cases are detected in Western countries with an incidence increasing every year, which correlates with population aging.

CRC development is a complex multi-factorial process occurring over many years as the result of an accumulation of genetic and epigenetic alterations in proto-oncogenes, tumor suppressor genes, and/or DNA repair genes, leading to transformation of normal colonic epithelium into glandular structures called adenocarcinomas (Fleming et al., 2012). The underlying causes of CRC are complex and heterogeneous. Both genetic and environmental factors can influence the initial steps and/or progression of CRC, which complicates the study of the disease etiology.

Depending on the origin of mutations, CRC can be classified as sporadic (70%) or inherited (30%). One key feature of both sporadic and familial CRC tumors is their high degree of genomic instability, arising from distinct molecular mechanisms defining different tumor molecular subtypes: (1) chromosomal instability (CIN), (2) microsatellite instability (MIS) resulting in hyper mutated tumors, (3) epigenetic instability with alteration in CpG island methylation (Muller et al., 2016). Despite this important molecular heterogeneity, defined signaling pathways are consistently altered in CRC tumors, including Wnt, TGF-beta, PI3K, RTK-RAS and P53 signaling (The Cancer Genome Atlas Network, 2012). Wnt pathway, which is crucial for intestinal epithelium homeostasis, is indeed constitutively activated in more than 90% of CRC tumors, and loss of function mutations in its negative regulator APC are found in 80% of non-hypermutated and 50% of hyper-mutated CRC tumors (The Cancer Genome Atlas Network, 2012).

In addition to genetic alterations, the tumor microenvironment plays a critical role in CRC development and important contributing factors are linked to nutrition, inflammation, epigenetics modifications and gut microbiota. The gut microbiota is currently considered as an organ which plays a crucial role in regulating host intestinal homeostasis through its capacity to modulate several biological processes including barrier, immunity and metabolic functions. A combination of external factors can influence microbial composition, including host genetics, diet, lifestyle, and environmental factors. These perturbations in the microbiota shift influence the balance between healthy and carcinogenesis. Alterations of the colon microbiota is recognized as an important player in the initiation and progression of CRC (Schwabe and Jobin, 2013 #337; Gagniere et al., 2016 #364; Braten et al., 2017; Flemer et al., 2017; Gao et al., 2017; Lucas et al., 2017). In addition, microbiota can modulate cancer therapy by its extensive metabolic capacity and profound immunomodulatory effects (reviewed by Pope et al., 2017 #462).

A critical question regarding CRC associated bacteria is whether they represent a consequence of altered host mucosal tissues or if the bacteria by themselves can have an oncogenic or pro-tumoral effect (Sears and Garrett, 2014; Raskov et al., 2017). Using microbiota transfer experiments in various CRC animal models, several studies have clearly shown that cancer-associated microbiota plays a role in cancer progression (Couturier-Maillard et al., 2013). Depletion of the intestinal bacterial microbiota in mice using antibiotics reduces the risk of colon cancer (Schwabe and Jobin, 2013 #337). Several molecular mechanisms have been proposed to explain bacteria-induced pro-tumoral effects: (i) induction of chronic inflammation; (ii) bacterial transformation of host metabolites into carcinogens; (iii) expression of specific bacterial factors such as toxins endowed with oncogenic properties, (iv) barrier failure (Schwabe and Jobin, 2013 #337; Gagniere et al., 2016 #364). A few bacterial species have been identified as playing a role in colorectal carcinogenesis such as *Fusobacterium nucleatum (Fn)*, enterotoxigenic *Bacteroides fragilis (ETBF)*, colibactin- and genotoxin-producing *Escherichia coli, Enterococcus faecalis, Clostridium septicum* (Gagniere et al., 2016 #364).

Both *Fn* and *ETBF* were shown to alter the Wnt/β-catenin signaling pathway. *F. nucleatum* was shown to adhere to and invade colonic cells through its unique surface adhesin FadA (Rubinstein et al., 2013). FadA binds to the host cell receptor E-cadherin promoting attachment and invasion of epithelial cells by Fn. FadA binding to E-cadherin leads to activation of β-catenin signaling (Bryant and Stow, 2004), resulting in increased cell proliferation (Rubinstein et al., 2013). *ETBF* are prevalent in the colon mucosa of CRC patients and able to modulate the mucosal immune responses and to induce epithelial cell changes (Sears et al., 2014; Boleij et al., 2015; Purcell et al., 2017). It was shown that *ETBF* secrete a zinc-dependent metalloprotease toxin called BFT which cleaves E-cadherin, thus causing nuclear translocation of β-catenin, increased c-Myc expression and cell proliferation (Wu et al., 2003). Of note, *Helicobacter pylori*, which is the sole bacterium clearly responsible for gastric cancer development, activates the β-catenin pathway resulting in increased cell proliferation (Parsonnet et al., 1991; Franco et al., 2005). *H. pylori* strain specific CagA protein is translocated into the host cell cytoplasm by a type IV secretion pilus where it interacts with tyrosine kinase c-Met receptor and its co-receptor CD44, leading to β-catenin activation and cellular proliferation (Suzuki et al., 2009; Bertaux-Skeirik et al., 2015). Besides CagA, several other mechanisms leading to β-catenin activation have been described in *H. pylori* altering the expression of Wnt ligands (Kirikoshi et al., 2001), activating Wnt receptors (Gnad et al., 2010), suppressing GSK3β (Sokolova et al., 2008; Nakayama et al., 2009), interfering with β-catenin/TCF4 complex by down-regulating the gastric tumor suppressor Runx3 (Liu et al., 2012), and interacting with E-cadherin to disrupt the E-cadherin/β-catenin complex (Murata-Kamiya et al., 2007) highlighting the importance of this signaling pathway in cancer development.

### Epidemiological Association Between CRC and *S. gallolyticus* subsp. *gallolyticus*

*Sgg* is an important cause of endocarditis, an inflammation of the inner layer of the heart (the endocardium) (Hoen et al., 2002). A relationship between *Sgg*-induced endocarditis and CRC was established for the first time by McCoy and Mason (1951). Later on, several epidemiological studies confirmed this association ranging from 47 to 85% between *Sgg* and CRC, depending on the techniques used for *Sgg* detection (Klein et al., 1977; Waisberg et al., 2002; Kok et al., 2007; Gupta et al., 2010; Corredoira et al., 2015; Chand et al., 2016). Most of these studies were performed on a selected cohort of patients with a history of *Sgg* bacteremia/endocarditis. A recent molecular analysis of tumoral and adjacent normal tissues from unselected CRC patients by quantitative PCR using *Sgg*-specific primers showed that about 74% of tumor tissue and 47% of adjacent normal tissues were positive to *Sgg* (Kumar et al., 2017). In striking contrast, another study on unselected CRC patients showed a much lower prevalence using quantitative real-time PCR. Only 6 out of 190 patients included (3.2%) were positive for *Sgg* (Andres-Franch et al., 2017). Nevertheless, the six positive cases were all from tumor tissue samples, while none of the normal mucosa samples presented *Sgg* DNA (Andres-Franch et al., 2017). These contradictory results either result from differences in the sampled population, but more likely from differences in the methodology used to detect *Sgg* (site of detection, number of samples, sample processing, conservation, enrichment of *Sgg* in specific medium, primers, qPCR techniques).

**FIGURE 1:**
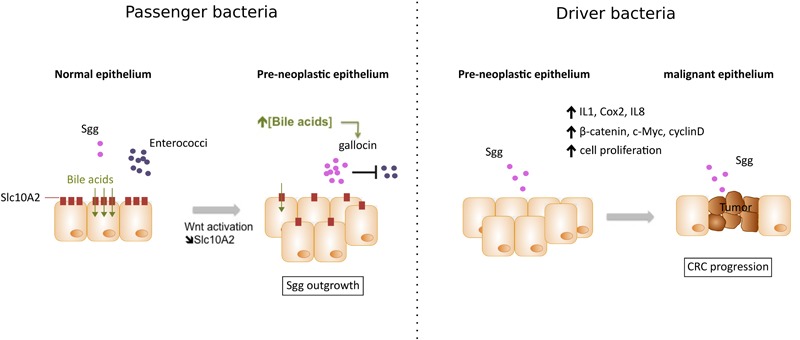
Two working models explaining *Sgg* association with colorectal cancer (CRC). (1) *Sgg* as a passenger bacterium: In pre-neoplastic epithelium, activation of the Wnt pathway leads to the downregulation of bile acids transporter Slc10A2 resulting in accumulation of bile acids- which in turn activates a specific “bacteriocin” enabling Sgg to kill related commensals (e.g., Enterococci). This local microbial imbalance can contribute to the development of CRC. (2) *Sgg* as a driver bacterium: High colonization of Sgg in pre-malignant epithelium can induce specific inflammatory responses (IL-1, COX-2, and IL-8) and increased cell proliferation associated with upregulation of β-catenin levels and its oncogenic downstream targets (c-Myc and cyclin D), thus accelerating transformation from pre-malignant to malignant epithelium.

A recent comparison of colorectal neoplasms associated to *Clostridium septicum (Cs)* to *Sgg* showed several differences in clinical presentation, underlying conditions, prognosis and long-term follow-up (Corredoira et al., 2017). *Sgg* positive cases were associated with advanced (52.3% vs. 5.2% for *Cs*) and non-advanced adenomas (28.1% vs. 0% for *Cs*) and the tumor was located mostly in the distal colon (65.6%) but also in the cecum/ascending colon (23.4%) and transverse colon (10.9%). In contrast, *Cs* positive cases were mostly associated with advanced neoplasia/invasive carcinoma (94.7% vs. 19.5% for *Sgg*) that were mostly located in the cecum/ascending colon (73.7%). These differences suggest that each bacterium (here *Cs* or *Sgg*) affects differently tumor development and that *Sgg* may play a role at the very early steps of CRC development. This hypothesis is in line with earlier observation that the majority of patients with *Sgg* positive endocarditis had asymptomatic colorectal tumors that were occasionally benign adenomas (Klein et al., 1977). Since the publication of the first complete genome of *Sgg* strain UCN34 (Rusniok et al., 2010), molecular studies were undertaken to determine if *Sgg* is a cause or a consequence of CRC. It is also possible for both scenarios to co-exist (**Figure 1**).

### *Sgg* as a Promoter of Colorectal Cancer

In favor of an etiological role of *Sgg* in CRC, the first experimental evidence that *S. bovis* could accelerate cancer development was reported in AOM-treated rats using *S. bovis* strain NCTC 8133 through increased inflammatory pathways (Ellmerich et al., 2000). However, following revision of *S. bovis* classification the strain NCTC 8133 was shown to belong to *S. bovis* type II/I now renamed *S. infantarius* (Biarc et al., 2004). In 2010, a molecular study demonstrating a significantly higher detection of *Sgg* in human neoplastic tissues versus normal adjacent tissue from the same patient was published (Abdulamir et al., 2010). It showed that bacterial colonization was accompanied by mRNA increase of genes encoding inflammatory molecules such as IL-1, COX-2, and IL-8; The authors proposed that this specific inflammatory response may drive the development of CRC (Abdulamir et al., 2010). A very recent study demonstrated that *Sgg* strain TX20005 promotes colorectal tumor development through increase of epithelial cell proliferation (Kumar et al., 2017). Using *in vitro* cell lines, Kumar et al. (2017) first showed that *Sgg* TX20005 increased cell proliferation in the following human colon cancer cell lines: HCT116, HT29, LoVo, but not in other colonic cell lines such as SW480, SW1116, or normal colonic cells CCD 841 CoN, FHC. Increased proliferation in responsive cell lines was associated with upregulation of β-catenin levels and its oncogenic downstream targets (c-Myc, cyclin D). Furthermore, using murine models, Kumar et al. (2017) showed that HCT116 cells cultured *in vitro* with *Sgg* and then injected into nude mice developed larger tumors as compared to control mice injected with HCTT16 co-cultured with non-pathogenic *Lactococcus lactis* MG1363. Secondly, in AOM-induced murine model of CRC, mice orally treated with *Sgg* displayed higher number of tumors, higher level of dysplasia, increased cell proliferation and β-catenin level in colon crypts as compared to control mice treated with *L. lactis* bacteria (Kumar et al., 2017). Interestingly, a preeminent early mutation in CRC is in the tumor suppressor gene APC found in 80% of human sporadic colon cancers and also responsible for the familial adenomatous polyposis syndrome, one of the main forms of hereditary colon cancer. Apc loss leads to the constitutive activation of Wnt/β-catenin pathway, which in turn induces cell proliferation and rapid loss of epithelial differentiation (reviewed in Zhan et al., 2017). Future studies will certainly aim at unraveling the molecular bases of *Sgg*-induced up-regulation of β-catenin in responsive cells.

### *Sgg* Benefits From the Tumor Microenvironment

Consistent with intestinal dysbiosis reports in CRC, metabolomic studies revealed that CRC microenvironment is strongly altered when compared to normal mucosal environment (Hirayama et al., 2009). Key features indicate a drastic decrease in glucose and pyruvate levels and an increase in lactate (low pH), amino acids, lipids, and fatty acids. Growth of Sgg in spent media of human malignant colonic cells (Caco-2 and HCT116) was investigated and compared to other intestinal bacteria. It was shown that particular metabolites derived from increased glycolysis in tumor cells, such as F6P, 3PG or alanine, benefit to Sgg for its own multiplication (Boleij et al., 2012a). In line with these findings, *S. bovis* was found to be one of the most efficient bacteria to utilize glucose in an experimental human *in vitro* gut fermentation model (Egert et al., 2007).

In addition, it is tempting to hypothesize that interactions mediated by both Pil3 and Pil1 with colonic mucins expressed in tumors such as MUC5AC and collagen type IV, respectively, can further increase *Sgg* preferential colonization of dysplastic tissues within the colon.

Finally, we recently showed that *Sgg* strain UCN34 is able to produce a specific bacteriocin, named gallocin, which contributes to enhance bacterial colon colonization in tumor bearing mice (Aymeric et al., 2017). It was shown that gallocin is able to inhibit the growth of closely related Enterococci commensals, thus creating an appropriate colonization niche for *Sgg*. Gallocin activity is strongly potentiated in the presence of secondary bile acids such as deoxycholic and lithocholic acids, which are known risk factors for CRC. By comparing Apc^+/-^ mice and their WT counterparts the authors showed that the presence of intestinal polyps *per se*, was sufficient to enhance *Sgg* UCN34 colonization in a gallocin-dependent manner. Indeed, following a one shot inoculation, *Sgg* UCN34 persisted for 3 months in adenoma-bearing host, whereas it was progressively excluded from the gut of healthy mice. This colonization advantage was lost with the gallocin-deficient mutant. The authors further unraveled a new link between Wnt pathway activation, an early step in CRC development, and increased luminal concentration of secondary bile acids by showing that Wnt activation resulted in decreased expression of the apical bile acids transporter Slc10A2 and reduced luminal bile acids reabsorption. Apc mutation, increased carcinogenic secondary bile acids and SGG colonization may thus be part of a vicious pro-tumoral triangle.

## Conclusion

For more than 50 years, clinical studies have strongly linked the presence of *Streptococcus bovis* biotype I, renamed *Streptococcus gallolyticus subsp. gallolyticus* (*Sgg*), to CRC. The first direct demonstration of the etiological role of *Sgg* isolate TX20005 in promoting CRC development was provided very recently (Kumar et al., 2017). But *Sgg* has also been shown to behave as a passenger bacterium benefiting from tumor metabolites (Boleij et al., 2012a) and able to secrete a specific “bacteriocin” that can kill closely related gut commensals (Aymeric et al., 2017) thus enabling a better colonization of murine colon in CRC-context. We thus conclude that Sgg is both a passenger and a cancer promoting bacterium (**Figure 1**). But in order to become a driver bacterium, *Sgg* first needs to colonize the colon and it does so only if pre-malignant conditions exist. So *Sgg* is not the principal cause of CRC but an auxiliary factor accelerating the development of CRC. Ultimately, the strong association of *Sgg* with CRC constitutes a solid argument to recommend a systematic colonoscopy for assessment of occult neoplasia in patients suffering of *Sgg* infections. Development of new molecular tools for the sensitive and specific detection of specific CRC-associated bacteria should help in the early detection of subclinical colonic lesions but may also add a weapon in the oncologists’ arsenal as demonstrated recently (Bullman et al., 2017; Yu et al., 2017). A future area of investigation will be to study the relationship between *Sgg* and host immunity, another important player in CRC development.

## Author Contributions

MM, EP-K, LA, and SD designed and wrote this review.

## Conflict of Interest Statement

The authors declare that the research was conducted in the absence of any commercial or financial relationships that could be construed as a potential conflict of interest. The handling Editor declared a past co-authorship with one of the authors SD.
